# The Relevance of Insulin Action in the Dopaminergic System

**DOI:** 10.3389/fnins.2019.00868

**Published:** 2019-08-16

**Authors:** Francesca Fiory, Giuseppe Perruolo, Ilaria Cimmino, Serena Cabaro, Francesca Chiara Pignalosa, Claudia Miele, Francesco Beguinot, Pietro Formisano, Francesco Oriente

**Affiliations:** ^1^Department of Translational Medicine, University of Naples Federico II, Naples, Italy; ^2^URT “Genomic of Diabetes,” Institute of Experimental Endocrinology and Oncology, National Research Council, Naples, Italy

**Keywords:** type 2 diabetes mellitus, insulin resistance, Parkinson’s disease, dopamine, neurodegeneration

## Abstract

The advances in medicine, together with lifestyle modifications, led to a rising life expectancy. Unfortunately, however, aging is accompanied by an alarming boost of age-associated chronic pathologies, including neurodegenerative and metabolic diseases. Interestingly, a non-negligible interplay between alterations of glucose homeostasis and brain dysfunction has clearly emerged. In particular, epidemiological studies have pointed out a possible association between Type 2 Diabetes (T2D) and Parkinson’s Disease (PD). Insulin resistance, one of the major hallmark for etiology of T2D, has a detrimental influence on PD, negatively affecting PD phenotype, accelerating its progression and worsening cognitive impairment. This review aims to provide an exhaustive analysis of the most recent evidences supporting the key role of insulin resistance in PD pathogenesis. It will focus on the relevance of insulin in the brain, working as pro-survival neurotrophic factor and as a master regulator of neuronal mitochondrial function and oxidative stress. Insulin action as a modulator of dopamine signaling and of alpha-synuclein degradation will be described in details, too. The intriguing idea that shared deregulated pathogenic pathways represent a link between PD and insulin resistance has clinical and therapeutic implications. Thus, ongoing studies about the promising healing potential of common antidiabetic drugs such as metformin, exenatide, DPP IV inhibitors, thiazolidinediones and bromocriptine, will be summarized and the rationale for their use to decelerate neurodegeneration will be critically assessed.

## Introduction

The prevalence of aging-associated chronic pathologies, such as neurodegenerative and metabolic diseases, has dramatically increased along with life expectancy (Zochodne and Malik, 2014). Type 2 Diabetes (T2D) embraces almost 90% of all cases of diabetes and actually represents a major public health problem worldwide. Chronic hyperglycemia is the hallmark of T2D, resulting from insulin resistance and beta cell dysfunction, and is associated with long-term complications, including retinopathy, nephropathy, micro and macro vascular diseases. More recently, experimental, clinical and neuroimaging data provided evidence of a connection between T2D and brain injury (Hamed, 2017). T2D-associated brain injury is largely linked to hyperglycemia and involves several different pathological events, such as oxidative stress (Muriach et al., 2014), mitochondrial dysfunction (Shokrzadeh et al., 2018), neuroinflammation (Rom et al., 2018), decrease of neurotrophins (Franco-Robles et al., 2014), modification of neurotransmitters (Datusalia and Sharma, 2014), vascular derangements (Kooistra et al., 2013), amyloid β deposition (Wang X. et al., 2014), increased tau phosphorylation (Platt et al., 2016) and progressive cognitive dysfunction (Simo et al., 2017). Epidemiological studies also support the evidence of a crosstalk linking T2D and neurodegenerative disorders (Morsi et al., 2018). In particular, an interesting association between T2D and PD has recently emerged from clinical, experimental and genome-wide association studies (Biosa et al., 2018; De Pablo-Fernandez et al., 2018).

Deterioration of dopaminergic neurons in the extrapyramidal tract of the midbrain is the trigger for PD pathogenesis, resulting from an interplay of genetic and environmental factors (Olanow et al., 2009). Most of the time PD is a sporadic disease, but few cases have genetic origin and several genes associated to PD have been found (Hernandez et al., 2016). Impairment of the dopaminergic neurons leads to a reduction in dopamine signaling and may lead to a relative increase in acetylcholine release from cholinergic neurons in the striatum, thereby contributing to dyskinesia (Heumann et al., 2014). Other typical motor symptoms of PD are bradykinesia, resting tremor, muscular rigidity and abnormal posture and gait (Olanow et al., 2009). In many cases of PD, loss of dopaminergic neurons in the substantia nigra is accompanied by the formation of intracellular neuronal inclusions composed of alpha-synuclein, known as Lewy bodies, in the central, autonomic, and peripheral nervous system. The diagnosis of PD is essentially based on the neurological examination, aimed to identification of characteristic motor signs, deriving from the loss of nigral dopaminergic neurons. The presence of a sustained response to dopamine drugs (dopamine agonists or levodopa) is also commonly used in diagnosis. Several non-motor symptoms are associated to PD, too. They include hyposmia, sleep behavior disorder, loss of olfaction, constipation, depression and global cognitive decline and precede the clinical effects of dopamine deficiency, sometimes for several years (Schapira et al., 2017). Unlike motor symptoms, non-motor symptoms of PD are not improved by dopamine replacement therapy and seem to derive from the formation of Lewy bodies beyond midbrain dopaminergic neurons (Dickson et al., 2009). Cognitive impairment and dementia are the most disabling non-motor symptoms of PD, resulting from microvascular disease (Kim J.S. et al., 2014), deposition of Lewy bodies in neocortical and limbic areas, hyperphosphorylated tau-containing neurofibrillary tangles and formation of amyloid-beta-peptide plaques (Irwin et al., 2013).

The onset of diabetes appears to increase severity of symptoms in PD patients (Sandyk, 1993), and epidemiological studies suggest that diabetes is a risk factor for PD (Hu et al., 2007; Cereda et al., 2012). Several studies have tried to explain how T2D affects pathogenesis and progression of PD. In 1993, Sandyk (1993) found a relationship between PD and T2D, evidencing that up to 50–80% of patients with PD featured an altered glucose tolerance in response to a glucose load. Some years later, Schernhammer et al. (2011) evaluated a population of 1.931 cases and 9.651 controls, evidencing a 36% increased risk of developing PD among patients with T2D. Similarly, a major risk of developing PD among individuals with T2D was found in the study conducted by Sun et al. (2012). In this case-control study, by examining a Chinese population of 603.416 diabetics and comparing it with a diabetes-free control, they found that diabetic women had a higher incidence of PD compared to men. Moreover, young diabetic men aged 21–40 years or diabetic women aged 41–60 years were more susceptible to the risk of Parkinsonism. Additional studies have suggested a positive association between PD risk and T2D. In particular, Hu et al. (2007) have studied a Finnish population of 51.552 individuals, both men and women, aged between 25 and 74, without a history of PD at baseline, concluding that T2D is associated with an increased risk of PD. Very recently, De Pablo-Fernandez et al. (2018) have found an association between diabetes and PD in a retrospective study, where a cohort of 2,017,115 individuals admitted for hospital treatment with a codified diagnosis of type 2 diabetes was compared with a reference cohort of 6,173,208 people without diabetes.

Nevertheless, there is also opposite evidence, pointing out a lower risk of PD incidence in subjects with T2D (Powers et al., 2006) and an inverse association of hyperglycemia with the onset of PD in individuals without any neurodegenerative disease (Miyake et al., 2010). These conflicting results could be due to confounding sampling of the different populations. For instance, in the report performed by Miyake et al. (2010), T2D diagnosis is based on the filling up of self-reported questionnaires. An additional source of confusion may be that the considered populations are too small to obtain significant results. Differences in study design and methodology and the difficulty to rule out confounders (such as microvascular damage and diabetic treatment) as risk factors for PD negatively affect data reproducibility, too. However, notwithstanding the heterogeneity of the data, the existence of a positive association between T2D and PD has been recently supported by interventional studies showing a reduction in incidence of PD in T2D patients treated with antidiabetic drugs such as metformin, sulfonylureas and exenatide, which exert neuro-protection (Wahlqvist et al., 2012; Aviles-Olmos et al., 2013). Several lines of evidence suggest that impairment of insulin signaling increase the risk of PD (Morris et al., 2008; Bosco et al., 2012; Ashraghi et al., 2016; Pang et al., 2016). Indeed, it has been recently found that insulin resistance, the impaired responsiveness to insulin, typical of T2D, occurs in PD brains and plays a key role in the progressive development of PD pathological hallmarks. In this review, we examine the relevance of insulin signaling in brain, especially for dopaminergic function, the relationship between insulin resistance and PD and finally we give an overview of the rationale underlying the use of drugs currently used for T2D in PD patients.

## Insulin Signaling in Brain

Insulin is a peptide hormone secreted in response to postprandial hyperglycemia from pancreatic beta−cells in blood circulation. Historically, insulin was essentially known as the main regulator of peripheral glucose homeostasis, since it induces glucose uptake in adipose tissue and skeletal muscle and glycogen synthesis in the liver, inhibiting in parallel hepatic glycogenolysis and gluconeogenesis (Haeusler et al., 2018).

In addition to these peripheral targets, insulin also undertakes a neuroregulatory function, although the physiological significance of its role in the brain has only recently started to emerge in both murine models and humans (Schubert et al., 2004; Duarte et al., 2012; Grote and Wright, 2016). Detectable concentrations of insulin have been found in several brain regions, including hypothalamus, olfactory bulb and midbrain since many years (Baskin et al., 1983), but it is not yet clear whether insulin is locally produced in CNS. Experimental evidence supports the hypothesis of insulin biosynthesis in adult neuronal cells derived from the hippocampus and olfactory bulb (Kuwabara et al., 2011) and by pyramidal neurons in the cortex (Dorn et al., 1982). Immunoreactive insulin and C-peptide were found in the brain from human cadavers, and, *in situ* hybridization showed the presence of insulin mRNA in the periventricular nucleus of the rat hypothalamus (Blazquez et al., 2014). Furthermore, Havrankova et al. (1978) showed the presence of insulin in rat brain at concentrations between 10 and 100 times higher than that in plasma. On the contrary, other studies did not confirm these results, and conclusive evidence for significant amounts of insulin synthesized in brain is lacking (Gray et al., 2014). However, insulin may enter brain parenchyma and precapillary space via a receptor-mediated transport (Duffy and Pardridge, 1987; Banks et al., 1997). Studies performed in an experimental model of human blood brain barrier (BBB) formed by isolated capillaries deriving from fresh human brain autopsy have shown that BBB insulin receptor has physicochemical properties similar to the IRs present in peripheral tissues such as adipocytes and hepatocytes (Pardridge et al., 1985; Plata-Salaman, 1991). Insulin transport to the CNS is reduced in high-fat diet-induced obesity (Kaiyala et al., 2000) and suppressed by hyperglycemia (Banks et al., 1997). In addition, Alzheimer’s disease and aging are associated with a reduction in insulin transport across the BBB (Craft et al., 1998; Frolich et al., 1998). Several studies have been performed in order to assess the integrity of BBB in PD although the results are still unclear. The observation that peripheral decarboxylase inhibitors, such as carbidopa and benserazide, do not reduce levodopa efficacy in brain indicate that BBB integrity is not compromised in parkinsonian patients (Rinne and Molsa, 1979). In support of this hypothesis, current and future therapeutic strategies for PD treatment are based on lipophilic substances or on a direct injection of proteins, genes and cellular therapies into the brain (Christine et al., 2009). Nevertheless, recent studies have also indicated that BBB is damaged in PD patients. Indeed, compromised BBB integrity in the striatum has been observed in postmortem brain tissue from PD patients (Gray and Woulfe, 2015). Furthermore, Dohgu et al. have indicated that monomeric alpha-synuclein induces BBB dysfunction by activating pericytes which, in turn, release inflammatory mediators (Dohgu et al., 2019). In conclusion, it is not possible to establish if insulin resistance in the PD brain arise from altered insulin transport across BBB. Hopefully, in the next future, advances in imaging techniques will allow to more carefully identify the source of insulin in the brain.

Interestingly, Jimenez-Jimenez et al. have compared cerebrospinal fluid (CSF) insulin levels in PD patients and in healthy subjects without finding significant differences between them (Jimenez-Jimenez et al., 2000). In contrast, other experimental evidence has shown that non-diabetic PD patients have increased blood glucose after oral glucose tolerance test without the concomitant rise in insulin levels, probably due to an impaired adaptive insulin secretion (Marques et al., 2018). Thus, the relationship between CSF/brain and serum insulin levels in PD needs to be elucidated. However, the specific role of this hormone in the different brain areas remains undeniable. Indeed, insulin elicits its effects by binding a specific tyrosine kinase receptor, expressed in different brain regions (Plum et al., 2005), including dopaminergic neurons (Figlewicz et al., 2003; Konner et al., 2011). Glucose uptake into neurons is insulin independent, thus in the brain insulin signaling regulates olfaction, mood and memory (McNay et al., 2010; Ketterer et al., 2011; Aime et al., 2012; Kleinridders et al., 2014; Biessels and Reagan, 2015; Heni et al., 2015). In addition, acting on glucosensing neurons of the hypothalamus, insulin modulates peripheral metabolism, hepatic glucose output, food intake, body weight, lipolysis and white adipose tissue browning (Blazquez et al., 2014; Dodd et al., 2015).

## Regulation of Survival of Dopaminergic Neurons

Well-characterized insulin functions in the central nervous system are the regulation of apoptosis during neuronal development and the enhancing of neuronal survival. This is not surprising since insulin binding to its receptor (IR) activates several intracellular effectors relevant to cell survival, such as PI3K/Akt pathway. Insulin, indeed, negatively modulates the expression of pro-apoptotic proteins protecting embryonic retinal cells during development from cell death (Diaz et al., 1999). Regarding the increase of neuronal survival, it is known that insulin signaling rescues rat hippocampal cells in culture injured by oxygen or glucose deprivation (Mielke and Wang, 2005) and has neuroprotective effects on H2O2-induced toxicity of retinoic acid (RA)-differentiated SH-SY5Y cells (Ramalingam and Kim, 2014). During the pathogenesis of PD, characterized by death of dopaminergic neurons in the substantia nigra pars compacta, insulin pro-survival ability is particularly relevant and clearly emerged in studies performed in SH-SY5Y cells pretreated with the neurotoxin MPP+ and in animal models (Moroo et al., 1994). In this cellular model of experimental PD, insulin prevented cell death in a dose dependent manner. It inhibits MPP + -induced iNOS and ERK activation, lowering in turn nitric oxide release, reactive oxygen species (ROS), calcium ion influx and finally decreasing the ratio of Bax to Bcl-2 through activating anti-apoptotic PI3K/Akt/GSK3 pathways (Ramalingam and Kim, 2016b).

## Modulation of Alpha-Synuclein Expression and Aggregation

**FIGURE 1:**
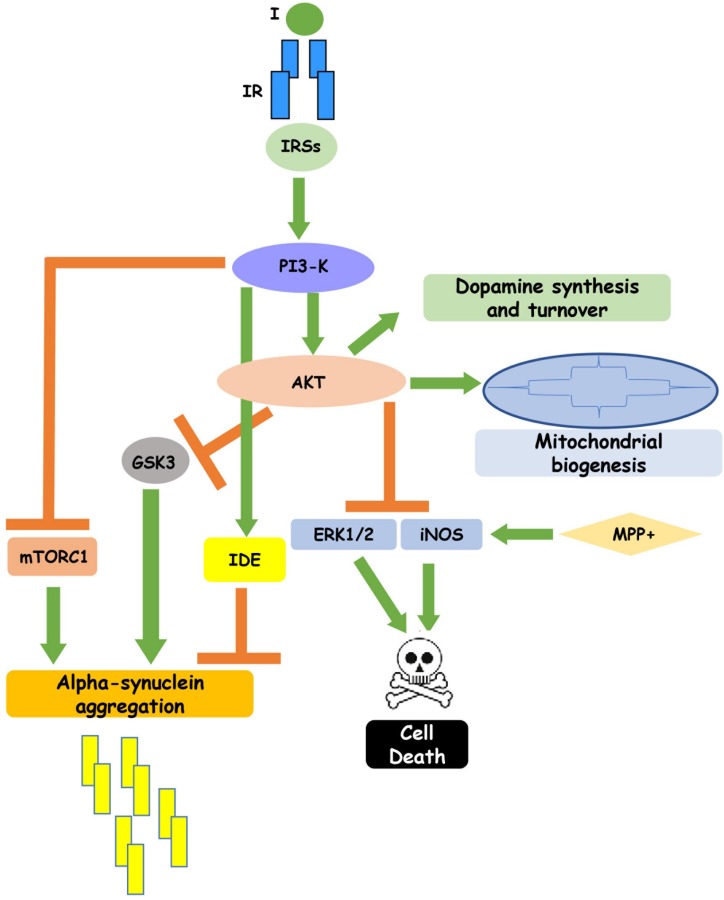
Insulin signaling regulates neuronal function. Insulin binding to its receptor, through the intracellular substrates IRSs, leads to activation of PI3-K pathway which, in turn, inhibits GSK3, mTORC1 and IDE, reducing alpha-synuclein aggregation, and enhances cell survival. In addition, insulin-induced PI3K activation stimulates dopamine synthesis and turnover and mitochondrial biogenesis. This figure includes experimental results obtained in cell cultures and partially confirmed in rodent and human brain.

Another characteristic neuropathologic feature in the PD brain is the accumulation of cytosolic inclusions of fibrillary forms of alpha-synuclein, called Lewy bodies. In C6 astrocytoma cells, a 24 h MPP + treatment induces a significant increase of a helically folded tetramer of alpha-synuclein accompanied by an augmentation of SNCA mRNA levels. Interestingly insulin affects alpha-synuclein expression and aggregation, too, by a mechanism involving the PI3K/Akt pathway (Ramalingam and Kim, 2017; Yang et al., 2018). Indeed, pretreatment with insulin induced a marked decrease in the tetrameric alpha-synuclein, preventing the cytotoxic effect of MPP + (Ramalingam and Kim, 2017). The molecular mechanisms underlying insulin protective action against MPP + neurotoxicity have been better clarified in SH-SY5Y cells, where insulin decreases alpha-synuclein and Cox-2 levels and blocks ROS-induced membrane damage. In parallel, it activates autophagy, integrins and syndecans signaling (Ramalingam and Kim, 2016a). Autophagy modulation by insulin is particularly relevant for PD pathogenesis, since it is crucial for elimination of abnormal and toxic protein aggregates. Insulin, indeed, blocking mTORC1 activity, stimulates autophagy of toxic proteins and activates Akt survival protein, through an mTORC2-mediated mechanism (Heras-Sandoval et al., 2014). The crucial importance of autophagy regulation by insulin is highlighted by the fact that the specific pharmacological inhibition of mTORC1 by rapamycin reduces alpha-synuclein aggregation (Sarkar et al., 2007) and prevents dopaminergic neuron loss (Tain et al., 2009). An additional plausible mechanism by which insulin promotes autophagy and negatively modulates alpha-synuclein toxicity is the inhibitory phosphorylation of GSK3beta by Akt. GSK3beta, indeed, co-localizes with alpha-synuclein in Lewy bodies and its expression is increased in postmortem brain from PD patients (Nagao and Hayashi, 2009) and in experimental models of PD associated with alpha-synuclein accumulation (Golpich et al., 2015). Recent evidences have revealed the presence of the microtubule associated protein tau in Lewy bodies, which is essentially known for its pathological role in Alzheimer disease, but it has recently been shown to participate in PD pathogenesis as well. GSK3beta inactivation by insulin is also involved in insulin-induced inhibition of tau phosphorylation which reduces neurotoxicity, increasing its binding to microtubules (Tokutake et al., 2012). Interestingly, insulin can directly affect alpha-synuclein turnover, reducing its aggregation and toxicity (Kao, 2009). Insulin action on alpha-synuclein aggregation is mediated by activation of IDE (insulin degrading enzyme), a highly conserved Zinc metallopeptidase which degrades amyloidogenic proteins. IDE, in turns, binds to alpha-synuclein oligomers, preventing them from further assembly into amyloid fibers that cause degeneration of dopaminergic neurons in PD patients (Sharma et al., 2015; Figure 1). Experiments performed in specific alpha-synuclein knockout mice have provided contrasting results. Indeed, while Rodriguez-Araujo et al. (2015) suggest that absence of alpha-synuclein in mice is associated with impairment in glucose metabolism during HFD-induced insulin-resistance, Geng et al. (2011) show an increased rate of insulin secretion in alpha-synuclein knockout mice, indicating alpha-synuclein as negative regulator of insulin secretion.

## Regulation of Mitochondrial Function and Inflammation

In addition to modulate alpha-synuclein amount, insulin is able to regulate mitochondrial biogenesis and to directly affect mitochondrial electron transport chain activity through stimulation of the IR/PI3K/Akt pathway, which suppresses FoxO1/HMOX1 induction (Cheng et al., 2010). Importantly, in hippocampal neurons, compounds activating IR also activate the AMPK-SIRT1-PGC1alpha signaling axis, enhancing in parallel mitochondrial function (Barhwal et al., 2015). Insulin’s ability to modulate mitochondrial membrane potential has long been characterized (Huang et al., 2003, 2005), but Aghanoori et al. (2017) recently revealed that insulin also controls mitochondrial function up-regulating mitochondrial electron transport system protein expression and complex activity.

Conversely, experimental models of insulin resistance feature altered levels of mitochondrial proteins in the substantia nigra (Khang et al., 2015), reduced levels of mitochondrial complex I and dysregulated calcium homeostasis (Moreira et al., 2006; Duarte et al., 2012). These phenomena impair mitochondrial biogenesis, inducing membrane depolarization and generation of excessive ROS, oxidative stress and increased cell death (Huang et al., 2003; Kleinridders et al., 2015). The link between mitochondrial dysfunction, insulin resistance and dopaminergic neuronal degeneration probably relies in the disruption of the Parkin-PARIS-PGC1alpha pathway. In chronic insulin resistance condition, indeed, reduced levels of Parkin have been observed in parallel with accumulation of a zinc finger protein, named PARIS, able to repress PGC1alpha expression and highly expressed in the substantia nigra of sporadic PD patients (Khang et al., 2015).

Insulin represents also a master regulator of extracellular events involved in PD pathogenesis, such as microglial activation and increase of pro-inflammatory mediators that contributes to ROS generation. Interestingly, several different pathways downstream IR activation such as PI3K/Akt and p38/MAPK pathways, are involved in TGF-beta1 neuroprotective effect against MPP + -induced neurodegeneration (Liu et al., 2016). Moreover, PI3K/Akt pathway decreases neuroinflammation up-regulating IkBalpha, a selective endogenous blocker of NF-kB, the main transcription factor responsible for expression of inflammatory genes (Khasnavis et al., 2012).

## Effect on Dopamine Synthesis and Turnover

Insulin itself represents also a physiological regulator of dopamine synthesis and clearance. A convincing demonstration for insulin relevance in modulation of dopamine signaling has been provided by the phenotype of NIRKO mice, featuring a neuron-specific knockout of IR. NIRKO mice, indeed, exhibit manifestations of anxiety and depressive-like behaviors. These hallmarks are accompanied by increased dopamine turnover, which in turn leads to decreased dopamine signaling in the striatum and nucleus accumbens. *In vitro* data indicate that in neuronal cells these alterations arise from a loss of insulin effect on expression of MAO A and MAO B, involved in inactivation of monoamine neurotransmitters (Kleinridders et al., 2015). Moreover, there is evidence in literature that insulin is able to regulate expression of tyrosine hydroxylase (TH), the rate-limiting step in the biosynthesis of dopamine. Insulin in rats was shown to induce a transient increase in TH mRNA in adrenal medulla (Rusnak et al., 1998; Xu et al., 2007). Conversely, pathological states characterized by impaired insulin signaling are associated with alterations of TH expression and/or activity. For instance, in experimental diabetes decreased TH activity in terminal fields for noradrenergic and dopaminergic neurons has been observed (Chu et al., 1986; Glanville and Anderson, 1986; Kono and Takada, 1994) and genetically diabetic Wistar rats show decreased immunoreactive TH (Nascimento et al., 2011). Moreover, in streptozotocin-treated rats, TH mRNA was increased in the locus coeruleus but decreased in the ventral tegmental area/substantia nigra pars compacta (Figlewicz et al., 1996). Part of the mechanism underlying TH modulation by insulin has been recently clarified in PC12 cells, where insulin regulates TH expression through the transcription factors HIF-1alpha and Nur77 (Fiory et al., 2018). These data have evidenced the critical role of insulin signaling in maintaining an appropriate dopaminergic tone by regulating TH expression in the central nervous system. In addition, studies in brain slices, in striatal synaptosomes, and *in vivo* have shown that insulin activation of IR increases dopamine uptake by the dopamine transporter (DAT). In particular, direct intracerebroventricular infusion of insulin results in increased DAT mRNA levels. Accordingly, when CNS insulin levels were reduced by 24- to 36-h food deprivation, DAT mRNA levels, assessed by *in situ* hybridization, were significantly decreased in the ventral tegmental area/substantia nigra pars compacta and the Vmax of dopamine uptake was significantly decreased in striatum from fasted rats. Interestingly, *in vitro* incubation with a physiological concentration of insulin augmented striatal dopamine uptake to control levels (Patterson et al., 1998). Similarly, insulin increases dopamine uptake and modulates DAT trafficking via PI3K in rat striatal synaptosomes (Carvelli et al., 2002). In particular, the key regulator downstream PI3K, responsible for DAT regulation by insulin, is Akt2 (Speed et al., 2010). These results suggest that synaptic dopamine signaling may be altered by reducing the available cell surface DATs in states of chronic hypoinsulinemia, such as diabetes (Carvelli et al., 2002). For instance, high fat feeding, impairs striatal insulin-induced activation of Akt, reducing in turns DAT cell surface expression and function and locomotor responses to amphetamine (Speed et al., 2011). Finally, it has been recently shown that insulin influences food choice amplifying action potential-dependent dopamine release in the nucleus accumbens and caudate-putamen through an indirect mechanism involving striatal cholinergic interneurons that express IR. Furthermore, the sensitivity of striatal dopamine release to insulin in rats is oppositely altered by chronic diet manipulations; indeed, food restriction enhances and obesogenic diet decreases responsiveness to insulin, respectively (Patel et al., 2018). On the other end, there is no known information about insulin-regulated food choice effect on PD onset and/or progression.

## Role in Cognitive Function

Insulin plays an acknowledged role in regulation of memory and cognitive function, too. This is particularly relevant for PD progression, since cognitive impairment represents a significant non-motor symptom of PD. PD patients, indeed, feature more rapid decline in cognitive domains and in memory (Aarsland et al., 2017), exhibiting a cognitive impairment which embraces a spectrum of severity from relatively mild symptoms to end-stage dementia (Davis and Racette, 2016). However, mild cognitive impairment can occur early in the course of PD, while dementia commonly characterizes advanced stages of PD (Hely et al., 2008). Interestingly, the prevalence of cognitive deficit is significantly higher in PD patients with diabetes mellitus than in patients with PD only, suggesting that diabetes may be one risk factor for cognitive dysfunction in PD patients (Yang et al., 2017). However, specific role of insulin in safeguarding cognitive function has been more clearly confirmed by studies showing that PD patients with dementia are prone to comorbid insulin resistance (Bosco et al., 2012; Ashraghi et al., 2016), even when they were unaffected by diabetes. Cognitive decline in PD and progression to dementia derive from alterations in hippocampal structure and function (Bouchard et al., 2008; Costa et al., 2012; Pan et al., 2013). This is plausible, since hippocampal neurons are particularly susceptible to alterations in insulin sensitivity (Fehm et al., 2006). Importantly, a high density of IRs has been found in the hippocampus, cortex and amygdala, where they participate in cognitive functions (Singh et al., 1997; Gerozissis, 2003). Furthermore, acute administration of insulin, through activation of hippocampal IRs, ameliorates performance on memory tasks in rats (Park et al., 2000) and enhances verbal memory and cognition in humans (Kern et al., 2001; Benedict et al., 2004). Insulin effects on cognition involves the PI3K/Akt pathway (McNay and Recknagel, 2011) and is probably mediated by its ability to affect synaptic plasticity. Activation of the PI3K/Akt pathway, indeed, maintains dendritic spine stabilization, necessary for memory consolidation (Goldin and Segal, 2003; Zhao and Townsend, 2009). The crucial insulin effector downstream PI3K/Akt pathway involved in preservation of cognitive function is GSK3beta. Insulin increases GSK3beta inhibitory phosphorylation through PI3K/Akt signaling. The phosphorylation of GSK3 beta, in turn, improves long-term memory in hippocampal-associated tasks, decreases tau and alpha-synuclein accumulation and neurotoxicity and reduces neuroinflammation and apoptosis. In conclusion, insulin alleviates cognitive impairment in PD via the inactivation of GSK3beta mediated by PI3K/Akt (Yang et al., 2018).

## Relevance of Insulin Resistance for Parkinson’s Disease

Interestingly, patients with PD feature augmented autoimmune reactivity to insulin (Wilhelm et al., 2007). Moreover, in the substantia nigra pars compacta of patients with PD, death of dopaminergic neurons is often anticipated by marked loss of IR mRNA and enhanced levels of IRS phosphorylation at serine residues, with inhibitory action on insulin signaling and subsequent increased insulin resistance (Moroo et al., 1994; Takahashi et al., 1996; Duarte et al., 2012; Morris et al., 2014). In particular, increased levels of IRS-1 pSer312 in the putamen and of pSer616 in hippocampal tissue of PD patients were found (Athauda and Foltynie, 2016). Likewise, both 6-OHDA-induced PD models and alpha-synuclein overexpressing mice show increased IRS phosphorylation at serine residues in the dopamine-depleted striatum (Morris et al., 2008, 2011a,b; Gao et al., 2015). In addition, increased nuclear translocation of PTEN and GSK3beta, paralleled by an impaired insulin signaling cascade, was observed in postmortem substantia nigra from PD patients (Sekar and Taghibiglou, 2018). Similarly, other authors have found decreased Akt phosphorylation in sections of substantia nigra from parkinsonian and control subjects (Malagelada et al., 2008; Timmons et al., 2009). These alterations may contribute to the pathogenesis and/or progression of PD. However, all of these results have been obtained in absence of “*ex vivo*” stimulation with insulin and, at the best of our knowledge, there is no evidence about the ability of PD postmortem brains to respond to insulin. Thus, the physiological decline in insulin signaling, which represents a typical hallmark of aging (Zhao et al., 2004; Kushner, 2013), is clearly accelerated in PD. On the other hand, the alterations of insulin signaling exacerbate PD clinical-pathological symptoms, enhancing dopaminergic degeneration and worsening disease progression and, in parallel, both motor and cognitive decline (Papapetropoulos et al., 2004). Several studies performed in animal models confirmed the onset of this deleterious crosstalk between insulin resistance and PD. In 2014, Wang and collaborators highlighted the relevance of insulin resistance for PD etiology using ob/ob and db/db mice as T2D model. These mice show insulin signaling impairment, ER stress and inflammation not only in peripheral tissue, but also in midbrain. It is worth of notice that they feature accumulation of alpha-synuclein and microglia activation along with increased production of pro-inflammatory cytokines. All these events were shown to enhance the vulnerability of dopaminergic neurons to MPTP neurotoxicity in the substantia nigra of db/db mice (Wang L. et al., 2014; Wang S. et al., 2014). Similar results were obtained in mice become insulin resistant upon a high-fat diet (HFD), which are more susceptible to PD inducing toxins, such as 6-OHDA and MPTP, characterized by a significant increase in nigrostriatal neurodegeneration and by a reduced dopaminergic signaling. This leads to a more severe motor deficits compared to matched controls (Choi et al., 2005; Morris et al., 2010, 2011a,b).

Recently, Sharma and Taliyan (2018) standardized an animal model suitable to mimic the comorbidity between insulin resistance and PD. To this aim, male Wistar rats were administrated 6-OHDA in medial forebrain bundle after 8 weeks feeding with high fat diet. The phenotype of these rats confirmed the capacity of insulin resistance to exacerbate PD pathology. In HFD-fed rats, indeed, 6-OHDA induced more pronounced neuronal damage and loss of striatal dopamine, leading, in parallel, to worst performance in behavioral tasks such as rotarod, narrow beam walk test and locomotor activity, compared to rats fed with standard diet.

The relevance of insulin resistance for PD has been further confirmed by the phenotype of transgenic mice overexpressing PED/PEA-15, a scaffold protein highly expressed in the brain and overexpressed in T2D subjects. These insulin resistant mice, indeed, show loss of dopaminergic neurons in the striatum and hypokinetic movements resembling PD motor alterations (Perruolo et al., 2016). Not least, NIRKO mice with neuron-specific IR knockout are the proof that insulin resistance is involved also in the onset of PD non-motor symptoms, since these mice develop increased dopamine turnover responsible for anxiety and depressive behaviors (Kleinridders et al., 2015).

Surprisingly, several studies found that alpha-synuclein increases inhibitory phosphorylation of IRS at serine residues, negatively regulating insulin signaling (Gao et al., 2015). Different mechanisms have been proposed to explain the deleterious effect of alpha-synuclein on insulin signaling. First, alpha-synuclein increases degradation of IRS-1, inhibiting protein phosphatase 2A through mTORC1 activation (Gao et al., 2015). In addition, alpha-synuclein induces microglial production of pro-inflammatory cytokines (Beraud et al., 2013; Blandini, 2013; Gallegos et al., 2015).

Overproduction of pro-inflammatory cytokines such as TNF-alpha in the CSF and CNS of PD patients was, indeed, evidenced in postmortem studies (Reale et al., 2009a,b). Similarly, peripheral concentrations of IL-6, TNF-alpha, IL-1beta, IL-2, IL-10, and C-reactive protein in PD patients are significantly higher compared with age-matched controls (Qin et al., 2016). Moreover, among newly diagnosed PD patients, those with higher levels of pro-inflammatory markers feature lower cognitive assessment scores (MMSE) and more rapid motor decline (Williams-Gray et al., 2009). Pro-inflammatory cytokines are probably responsible for increased activity of IRS serine kinases such as JNK, involved in the onset of neuronal insulin resistance (Peng and Andersen, 2003; Klintworth et al., 2007; Morris et al., 2008).

Interestingly, in HFD fed mice, restoring of IR signaling by inhibition of protein tyrosine phosphatase 1B or by treatment with the small molecule IR sensitizing agent, TCS 401, re-establishes insulin positive action on dopamine release and reuptake at dopamine terminals in the nucleus accumbens (Fordahl and Jones, 2017). Similarly, treatment with insulin sensitizing drugs and normalizing HFD ameliorate depressive behaviors in rodents (Yamada et al., 2011; Sharma et al., 2012). This evidence further suggests that insulin resistance could represent a common risk factor involved in both T2D and PD pathogenesis.

## Insulin Resistance Treatments in Parkinson’s Disease

Both insulin resistance and PD can be defined as multifactorial disorders due to the interaction of environmental factors with a genetic susceptibility. Thus, modification of lifestyle and health behaviors, such as diet, can improve and prevent the onset of these diseases. It is well established that the MD, a nutritional model widespread in some countries of the Mediterranean sea such as southern Italy, Spain and Greece, which is based on a relatively higher intake of cereals, fruit, vegetables, seeds, olive oil (unsaturated fat) compared to a more rare use of red meat and animal fats (saturated fats), has many beneficial effects on insulin-resistance and T2D (Giugliano and Esposito, 2008; Grosso et al., 2014; Garcia et al., 2016). More recently, some authors have shown that MD seems to play also a neuroprotective role, although not all of epidemiologic studies report a positive function of MD on neurodegenerative diseases and further studies are required to validate these evidences (Alcalay et al., 2012; Okubo et al., 2012; Martinez-Lapiscina et al., 2013a,b; Cassani et al., 2017; Table 1).

**TABLE 1:** Role of insulin resistance treatments in Parkinson’s disease.

**Insulin resistance treatments**	**Role in Parkinson’s disease**	**References**
Mediterranean-style diet	neuroprotection ?	Alcalay et al., 2012
		Okubo et al., 2012
		Martinez-Lapiscina et al., 2013a
		Martinez-Lapiscina et al., 2013b
		Cassani et al., 2017

Insulin	neuroprotection	Perlmuter et al., 1984
	memory improvement	Helkala et al., 1995
	↓ neuroinflammation	Vanhanen et al., 1999
	↓ oxidative stress	Benedict et al., 2004
		Pang et al., 2016
		Singh et al., 1997
		Gerozissis, 2003

Metformin	neuroprotection	Ng et al., 2012
		Perez-Revuelta et al., 2014
		Hsu et al., 2011
		Imfeld et al., 2012
		Moore et al., 2013
		Kuan et al., 2017

GLP-1 receptor agonists	neuroprotection	Li et al., 2009
	amelioration of motor function	Bertilsson et al., 2008
		Kim et al., 2009
		Chen et al., 2015
		Aviles-Olmos et al., 2013
		Aviles-Olmos et al., 2014
		MacConell et al., 2015
		Athauda et al., 2017

DPP-4 inhibitors	neuroprotection	Svenningsson et al., 2016
	↓ neuroinflammation	Matteucci and Giampietro, 2015
	↓ oxidative stress	Yazbeck et al., 2009
		Abdelsalam and Safar, 2015
		Nassar et al., 2015

Thiazoledinediones	neuroprotection ?	Rabchevsky et al., 2017
		Dehmer et al., 2004
		NINDS Exploratory Trials in Parkinson Disease (NET-PD) FS-ZONE Investigators, 2015
		Chang et al., 2015

**FIGURE 2:**
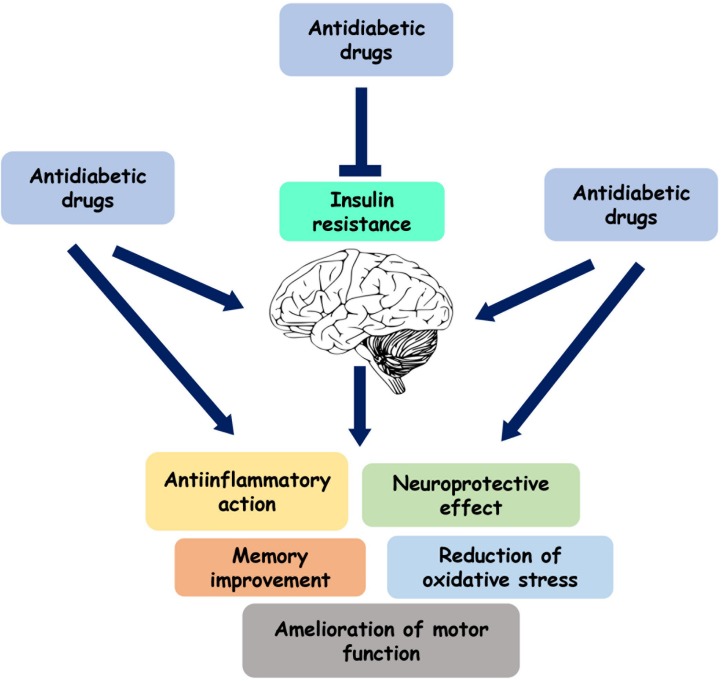
Role of the antidiabetic drugs on brain function. Several antihyperglycemic agents play a neuroprotective and anti-inflammatory role by directly acting on brain or by reducing insulin resistance.

In parallel with the healthy lifestyle, insulin and several drugs currently used for the treatment of insulin resistance have been suggested to have therapeutic effects in patients with PD (Figure 2 and Table 1). These substances include metformin, exenatide, thiazolidinediones, and bromocriptine.

The role of insulin in PD treatment has been firstly evidenced by several studies indicating the presence of frequent amnesic defects in T2D patients (Perlmuter et al., 1984; Helkala et al., 1995; Vanhanen et al., 1999). Subsequently, these observations have been confirmed by the fact that intranasal administration of insulin in the hippocampus can improve memory deficits in humans (Benedict et al., 2004). However, the importance of insulin is not limited to the learning and memory processes, but also extends to its ability to induce anti-inflammatory and neuro-protective responses. Indeed, intranasal insulin administration protects against substantia nigra dopaminergic neuronal loss and alleviates motor deficits induced by 6-OHDA in rats (Pang et al., 2016). The positive effects mediated by insulin can be probably related to the capacity of this hormone to improve brain oxidative stress, apoptosis, autophagy and neuroinflammation and to the presence of its receptor in the CNS, in particular in the hippocampus and medial temporal cortex and amygdala, as described before (Singh et al., 1997; Gerozissis, 2003).

Metformin belongs to the biguanide family and is the most frequently used oral antidiabetic drug (Viollet et al., 2012). Met, by activating AMPK or increasing the IR expression and tyrosine kinase activity (Musi et al., 2002; Viollet et al., 2012; Rena et al., 2017), reduces hepatic gluconeogenesis and increases insulin-stimulated glucose uptake in skeletal muscle and adipocytes. In addition, Met decreases free fatty acid oxidation, improving insulin sensitivity (Musi et al., 2002; Rena et al., 2017) and insulin secretion from pancreatic beta-cells (Sreenan et al., 1996). Further studies suggest that metformin can cross BBB and activate AMPK in the CNS (Nath et al., 2009). In addition, this drug has been shown to rescue dopaminergic dysfunction and mitochondrial abnormalities in Drosophila models of PD (Ng et al., 2012) and to reduce the phospho-Ser129 alpha-synuclein, the modified form of alpha-synuclein that occurs most frequently within PD, both *in vitro* and *in vivo* (Perez-Revuelta et al., 2014). Nevertheless, studies performed in humans revealed contrasting results. Indeed, while Hsu et al. have suggested that can reduce the risk of dementia (Hsu et al., 2011) in diabetic patients, other authors indicate that metformin exposure in patients with T2D may lead to the development of neuronal diseases, including dementia and PD (Imfeld et al., 2012; Moore et al., 2013; Kuan et al., 2017).

Glucagon-like peptide-1 (GLP-1) receptor agonists, by mimicking the effects of the incretin hormone GLP-1, increase glucose-mediated insulin secretion and reduce postprandial glucagon levels, gastric emptying rate, food intake and body weight. Differentially from GLP-1 hormone, having a short half-life, GLP-1 agonists have two important properties that include the longer duration of action after subcutaneous administration respect to GLP-1 and the resistance to degradation mediated by dipeptidyl-peptidase 4 (DPP-4) enzymes (Prasad-Reddy and Isaacs, 2015). Several GLP-1 receptor agonists, including lixisenatide, exenatide and liraglutide, induce neuroprotective effects, and, in particular, exenatide (Ex-4), a synthetic version of exendin 4, has been suggested to have an important role in PD. Indeed, both “*in vitro*” and “*in vivo*” studies have demonstrated the ability of exenatide to mediate neurotrophic and neuro-protective effects. In particular, Li et al. (2009) have shown that Ex-4 treatment protects dopaminergic neurons against degeneration, preserves dopamine levels and improves motor function in the MPTP mouse model of PD. Similar results have been obtained by Bertilsson et al. (2008) who suggest that that Ex-4 significantly increases the number of neurons positive for TH and vesicular MAO transporter 2 in the substantia nigra of animals lesioned with 6-OHDA. Several mechanisms by which exenatide protects form neurodegeneration have been hypothesized. Kim et al. (2009) have showed that this drug protects dopaminergic neurons by preventing MPTP-induced microglial activation and MMP-3 expression. Other authors have demonstrated that GLP-1 receptor stimulation reduces apoptosis by promoting Bcl-2 expression and inhibiting the activation of caspase 3 and preserves mitochondrial function in dopaminergic neurons (Chen et al., 2015). Toxin-based models of PD, despite their limited translational value, have allowed to clarify mechanisms of action of GLP-1 agonists. Nevertheless, it is still unclear which of the previously described pathways are crucial for the GLP-1 agonists therapeutic effects for PD (Foltynie and Athauda, 2018). Clinical trials have also validated the positive effects of GLP-1R agonists in PD, underlining the safety and tolerability of this drug (Aviles-Olmos et al., 2013, 2014; MacConell et al., 2015; Athauda et al., 2017). Nevertheless, the difficulty to compare each GLP-1R agonists under the same conditions limits, in part, the reproducibility of these studies.

DPP-4 is an enzyme which rapidly inactivates GLP-1 and GIP incretins, limiting their hypoglycemic action (Hansen et al., 1999). Furthermore, increased serological levels of DPP-4 have been observed in diabetic patients (Kim N.H. et al., 2014) and, thus, several DPP-4 inhibitors are used in the effective treatment of T2D. Treatment with DPP-4 inhibitors improves metabolism, insulin secretion and reduces glucagon secretion. Compared to GLP1 analogs, DPP-4 inhibitors are not able to induce weight loss, but in any case they do not lead to an increase in body weight, which instead occurs with sulfonylurea or insulin treatment.

Since the discovery of neurotrophic and immune regulating functions of DPP-4 inhibitors in the CNS, increasing studies supports the idea that DPP-4 might also be involved in the development of neurological disorders with a neuroinflammatory component.

Svenningsson et al. (2016) in a nationwide case-control study, found a significantly decreased incidence of PD among individuals with a record of DPP-4 inhibitor intake. The authors hypothesize that the this positive effect can be due not only to the increase of GLP-1/GLP-1R binding, but also by reducing the degradation of some neurotrophic neuropeptides, including pituitary adenylate cyclase-activating polypeptide (PACAP), substance P, neuropeptide Y, and gastrin-releasing peptide (Matteucci and Giampietro, 2015). Furthermore, DPP-4 inhibitors may have direct immunosuppressive effects, providing interesting insights for the future therapeutic development of treatments of neurological conditions with recognizable immune-related dysfunctions (Yazbeck et al., 2009; Svenningsson et al., 2016).

Other studies have shown the antiparkinsonian effect of vildagliptin, a dipeptidyl peptidase (DPP)-4 inhibitor, in rotenone−induced PD model in rats. Indeed, in these animals, vildagliptin by blocking the RAGE/NFκB cascade, suppresses inflammatory, oxidative stress, and apoptotic mediators reducing death of dopaminergic neurons and motor impairment (Abdelsalam and Safar, 2015). Similar results have been obtained by Nassar et al. (2015) using saxagliptin, another DPP-4 inhibitor.

Thiazoledinediones, that include rosiglitazone and pioglitazone, are oral hypoglycemic agents which bind and activate the nuclear receptor PPARγ. This protein is expressed not only in many insulin target tissues, but also in the substantia nigra and in the putamen nucleus (Swanson and Emborg, 2014). TZDs improve insulin-resistance in several ways, including the reduction of circulating fatty acids, the activation of the Glut4-mediated glucose transport and the decrease of the levels of inflammatory cytokines (Davidson et al., 2018).

Moreover, pioglitazone mediates its neuro protective effect, by binding a protein residing in the mitochondrial outer membrane, called MitoNEEt and regulating the activity of complex I in neuronal cells (Rabchevsky et al., 2017). In addition, this drug blocks the nitric oxide-mediated toxicity in MPTP-treated mice (Dehmer et al., 2004). As for the other anti-diabetic medications, studies performed in humans about the efficacy of TZDs in PD are still disappointing (NINDS Exploratory Trials in Parkinson Disease (NET-PD) FS-ZONE Investigators, 2015). A possible explanation for difficulty to obtain significant data in neurological disorder such as parkinsonism can be probably related to the poor capacity of TZDs to cross the BBB. Indeed, pioglitazone and rosiglitazone are substrates of the P-glycoprotein. This protein increases during the inflammatory state that occurs in PD and acts as a stereoselective barrier preventing the entry of TZDs into the brain (Chang et al., 2015).

A class of drugs capable of activating D2R dopaminergic receptors is represented by the dopaminergic agonists. In particular, an ergoline derivative, bromocriptine, indicated for the treatment of patients with parkinsonism who no longer respond to treatment with levodopa, improves glycemic homeostasis and is used in the treatment of T2D since 2009. The mechanism of action of bromocriptine is still not very clear. This drug, by activating D2 and blocking D1 receptors, is able to reduce blood glucose and serum triglycerides levels and to decrease body weight (Kalra et al., 2011; Lopez Vicchi et al., 2016). Furthermore, bromocriptine directly activates the alpha 2-adrenergic receptors, inhibiting glucose-stimulated insulin secretion in pancreatic beta cells (Kalra et al., 2011; Lopez Vicchi et al., 2016).

Studies performed on animal models, in particular on ob/ob mice and Syrian hamsters suggest that bromocriptine treatment improves obesity and associated metabolic dysfunctions and inhibits the seasonally occurring obesity, hyperinsulinemia, insulin resistance and impaired glucose tolerance (Cincotta and Meier, 1995; Liang et al., 1998; Luo et al., 1998). In addition, several clinical trials have demonstrated the beneficial effect of bromocriptine on glycemia and weight in obese non-diabetic and diabetic subjects (Meier et al., 1992; Pijl et al., 2000; Aminorroaya et al., 2004; Gaziano et al., 2010). Thus, despite bromocriptine has been used since the 1960s for treatment of PD, acromegaly and prolactinomas, only recently its relevance has been demonstrated in T2D, encouraging its future application.

From these data, it is clear that the correction of metabolic disorders is of fundamental importance in the care of PD. However, actually, there is no resolutive antihyperglycemic treatment able to improve PD, slow down its progression and alleviate its symptoms. Thus, the future challenge of the PD research aims to identify new molecules that are more effective and tolerable both in PD and in insulin-resistance than the traditional ones.

## Conclusion

Several clinical and experimental studies indicate a higher prevalence of PD in patients diagnosed with diabetes. Indeed, it is now clear that the loss of insulin signaling may cause neuronal mitochondrial dysfunction and oxidative stress followed by loss of dopaminergic neurons and impaired memory functioning. These results have been corroborated by studies performed in animal models and by the positive action that some antidiabetic drugs induce with significant benefits in patients diagnosed with PD. However, although the scientific research has reached several promising results, further and more detailed investigations are necessary to validate these studies in order to discover new therapeutic avenues.

## Author Contributions

FF and GP prepared the first draft of the manuscript. IC, SC, FP, and CM were involved in the literature search. FB and PF critically revised the manuscript. PF and FO supervised the work and wrote the final version of the manuscript.

## Conflict of Interest Statement

The authors declare that the research was conducted in the absence of any commercial or financial relationships that could be construed as a potential conflict of interest.

## Abbreviations

6-OHDA: 6-hydroxydopmin
AMPK: Adenosine Monophosphate-Activated Protein Kinase
BAX: (B Cell Lymphoma)-Associated X
BCL: B Cell Lymphoma
COX: Cyclooxygenase
DPP-4: dipeptidyl-peptidase IV
ERK: Extracellular Receptor Kinase
Ex-4: Exenatide
GLP: Glucagon-like peptide
GSK: Glycogen Synthase Kinase
HFD: high fat diet-treated
IL: Interleukin
iNOS: Inducible nitric oxide synthase
IR: Insulin receptor
IRS: Insulin receptor substrate
JNK: Jun N-terminal Kinase
MD: Mediterranean-style diet
Met: Metformin
MMP-3: matrix metalloprotease 3
MPP+: 1-methyl-4-phenyl pyridinium
MPTP: 1-methyl-4-phenyl-1,2,3,6-tetrahydropyridine
NIRKO: neuron-specific insulin receptor knockout
PD: Parkinson’s disease
PED/PEA: Phosphoprotein enriched in diabetes/phosphoprotein enriched in astrocytes
PGC-1 α: peroxisome proliferator-activated receptor gamma coactivator 1-alpha
PI3K: Phosphoinositide 3-kinases
PPAR γ: Peroxisome proliferator-activated receptor gamma
RA: Retinoic acid
ROS: Reactive oxygen species
T2DM: Type 2 Diabetes Mellitus
TORC: cytochrome c-type protein
TNF: Tumor Necrosis Factor
TZD: Thiazoledinediones.
